# Paddling Upstream With Point-of-Care Ultrasound to Diagnose Cardiac Ascites

**DOI:** 10.7759/cureus.11604

**Published:** 2020-11-21

**Authors:** Patricia C Cheung, Jason P Williams

**Affiliations:** 1 Internal Medicine, Emory University School of Medicine, Atlanta, USA; 2 Medicine, Atlanta Veterans Affairs Medical Center, Atlanta, USA; 3 Hospital Medicine, Emory University School of Medicine, Atlanta, USA

**Keywords:** point-of-care-ultrasound, portopulmonary hypertension, cirrhosis, cardiac ascites, right ventricular failure

## Abstract

Ascites has multiple etiologies, including cirrhosis and heart failure, which can be differentiated by point-of-care ultrasound (POCUS). One cause of cardiac ascites that can be difficult to identify is portopulmonary hypertension (PPH), a rare disorder caused by pulmonary artery vasoconstriction due to advanced liver disease. POCUS can readily identify right ventricular dysfunction which can accelerate a PPH diagnosis. This case report describes the use of POCUS to work-up new onset ascites and expedite diagnosis of cardiac ascites due to PPH.

## Introduction

Although cirrhosis is the most common cause of ascites in the United States, assessment of other etiologies, including cardiac ascites, is important for directing management [1]. Historically, the diagnosis of cardiac ascites requires a paracentesis to identify total protein values ≥2.5 g/dL in ascites fluid [1]. However, paracentesis is invasive and total protein is not always ordered as part of standard ascitic fluid analysis. Point-of-care ultrasound (POCUS) is noninvasive and can readily identify elevated central venous pressure (CVP) which should prompt measurement of ascites total protein and further cardiac imaging [2].

Rare causes of cardiac ascites such as portopulmonary hypertension (PPH) can be easily overlooked. Patients with PPH are often asymptomatic or have vague complaints such as weakness or fatigue [3-4]. As such, diagnosis may be delayed. Only patients recognized early in the disease course have a chance for cure: liver transplantation [3].

Point-of-care ultrasound has been increasingly used to aid diagnosis. It has vast applications in identifying pulmonary, cardiac, abdominal, and vascular diseases [2]. For patients presenting with dyspnea to the ED, use of POCUS decreases time to diagnosis without sacrificing accuracy [5]. To the best of our knowledge, this is the first case report describing the use of POCUS to diagnose PPH. We aim to describe a framework for POCUS to work up new onset ascites for emergency medicine, internal medicine, and gastroenterology physicians. This approach works its way upstream from ascites, to the inferior vena cava (IVC), and finally the heart.

## Case presentation

**Figure 1:**
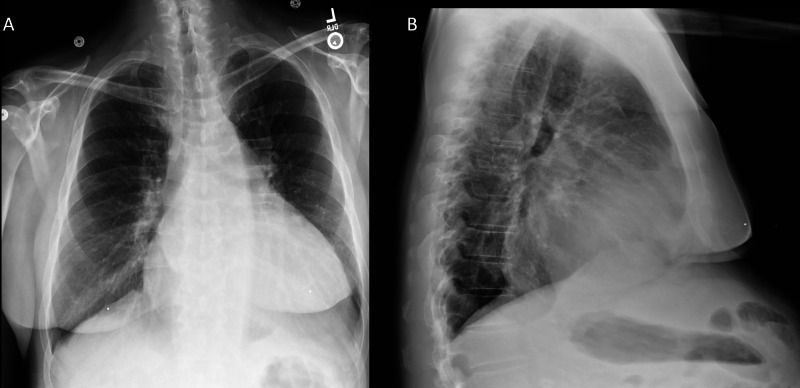
Chest X-Ray (A) Posterior-anterior chest X-ray with clear lung fields. (B) Lateral chest X-ray with clear lung fields.

A 53-year-old man with a history of congestive heart failure and alcohol use disorder presented to the ER with shortness of breath, abdominal distension, orthopnea, and a 20-pound weight gain in the prior two months. He reported that he had a 10 L paracentesis one year ago. He denied a history of coronary artery disease. The patient also reported that he used alcohol daily and was an active smoker. The patient’s home medications included carvedilol, furosemide, spironolactone, atorvastatin, metformin, and albuterol. The patient’s vital signs were within normal limits. Cardiac examination revealed a systolic murmur at the cardiac apex, however, jugular venous pulsation was unable to be observed because of body habitus. On pulmonary exam, the patient’s lungs were clear to auscultation in all lung fields. The patient had a distended abdomen with a positive fluid wave. The patient did not have lower extremity edema. His laboratory workup was notable for creatinine of 1 mg/dL, platelets of 33 x 103/mm3, INR of 1.1, albumin 3 g/dL, bilirubin 0.8 mg/dL, and pro-brain natriuretic peptide (BNP) of >5000 pg/mL. The chest X-ray was unremarkable (Figure 1). 

At this point the etiology of dyspnea was thought to be related to congestive heart failure or ascites. The history of congestive heart failure, elevated BNP, and orthopnea supported congestive heart failure as the etiology of his dyspnea, but he had a normal lung exam, a normal chest X-ray, and no lower extremity edema. Meanwhile, the abdominal distension, positive fluid wave, and history of a therapeutic paracentesis suggested the presences of marked ascites. Large volume ascites can compress the diaphragm and cause orthopnea and dyspnea. However, management of ascites and congestive heart differ, and it was important to accurately distinguish the two (Table 1) [1].

**Table 1:** Volume overload management.

	Ascites	Congestive heart failure
Acute fluid removal	Large volume paracentesis	Aggressive IV diuresis
Maintenance of euvolemia	Spironolactone:furosemide = 50:20	High dose furosemide

An attending with five years of POCUS experience then performed POCUS to further assess the patient. Abdominal ultrasound revealed only moderate ascites in the suprapubic region (Video 1A,B), but no other significant fluid pockets were found in the bilateral lower compartments. This moderate volume of ascites made diaphragmatic compression as the etiology of his dyspnea less likely. To further characterize the etiology of the ascites, the probe was moved to the subxiphoid position to identify the IVC. His IVC was distended more than 2.1 cm with less than 50% collapsibility consistent with an elevated CVP > 10 mmHg (Video 2A,B) [2]. In order to characterize the etiology of this high CVP, the probe was then moved to the parasternal position to assess for cardiac pathology. In the parasternal long axis view, the left ventricle demonstrated normal ejection fraction, but the right ventricle was severely dilated, and a moderate pericardial effusion was observed (Video 3A). In the parasternal short-axis view, the right ventricle was observed to be larger than the left ventricle, with septal flattening during diastole suggesting severe right ventricular dysfunction (Video 3B). 

**Video 1:** Ascites ultrasound. (A) Suprapubic ultrasound with ascites superior to the bladder. (B) Suprapubic ultrasound with 7.3 cm maximum depth of ascites.

**Video 2:** IVC ultrasound. (A) IVC in the longitudinal view with minimal respiratory collapse. (B) IVC in the longitudinal view with a dilated maximum diameter. IVC, inferior vena cava; RA, right atrium

**Video 3:** Cardiac ultrasound. (A) Parasternal short axis view of the heart with a moderate pericardial effusion and right ventricular dilation. The RV is larger that the Ao outflow tract and LA which violates the rule-of-thirds. (B). Parasternal short axis view demonstrates septal flattening (D shaped left ventricle) due to right ventricular pressure overload and a moderate pericardial effusion. Ao, aortic outflow tract; LA, left atrium; LV, left ventricle; RV, right ventricle

A paracentesis revealed 366 x 103/mm3 white blood cells with 61% monocytes and macrophages. The serum-ascites albumin gradient was 1.5, suggestive of portal hypertension. Finally, the ascites total protein was >2.5 g/dL, which confirmed the diagnosis of cardiac ascites. He was started on IV diuresis and had a 20-pound weight loss over the subsequent seven days with resolution of his shortness of breath and orthopnea.

The severe right heart dysfunction found on POCUS prompted a workup for pulmonary hypertension. A transthoracic echo confirmed the findings on POCUS including left ventricular ejection fraction of 55%-60%. There was moderate-to-severely dilated right ventricle with moderate-to-severe reduction in right ventricular systolic function. The right atrium was moderately dilated with moderate pulmonary hypertension (right ventricular systolic pressure = 60 mmHg). The IVC was dilated with less than 50% collapse with respiration, and a small pericardial effusion was seen. The patient underwent a right heart catheterization after aggressive IV diuresis which showed a right atrial pressure of 7 mmHg, pulmonary artery pressure of 73/26 mmHg, mean pulmonary artery pressure (mPAP) of 45 mmHg, and a pulmonary capillary wedge pressure of 13 mmHg, with a cardiac index of 2.7 L/min/m2 and pulmonary vascular resistance (PVR) of 498 dyn·s/cm5. His pulmonary hypertension workup included normal autoimmune serologies, normal diffusion on pulmonary function tests, normal high-resolution chest CT, and a normal V/Q scan. After ruling out secondary causes of pulmonary hypertension, he was diagnosed with Group 1 Pulmonary Arterial Hypertension due to PPH. He was enrolled into a sober living facility and is being followed closely by the liver transplant clinic.

## Discussion

**Figure 2:**
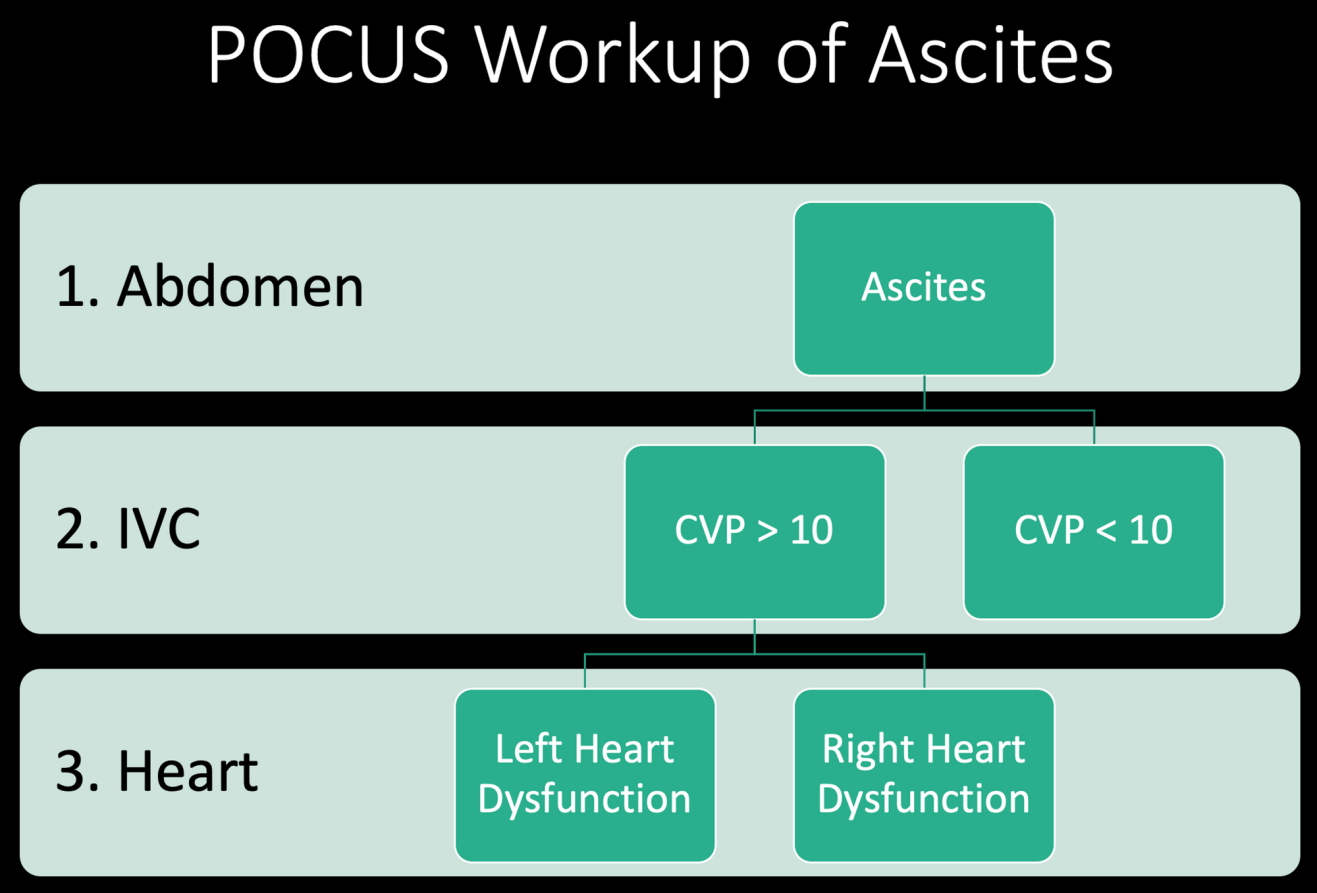
Framework for the workup of ascites. Step 1: confirm ascites. Step 2: evaluate the IVC. Step 3: If the CVP is elevated evaluate the heart. CVP, central venous pressure; IVC, inferior vena cava; POCUS, point-of-care ultrasound

This patient was originally thought to have decompensated alcoholic cirrhosis requiring a therapeutic paracentesis in order to resolve his dyspnea. A bedside ultrasound revealed a high CVP which was suspicious for a cardiac etiology of his ascites. This prompted a POCUS of his heart. The recognition of cor pulmonale prompted aggressive IV diuresis, and ultimately revealed a new diagnosis PPH. Working our way upstream from ascites, to the IVC, and finally the heart led to the diagnosis on hospital day one (Figure 2). 

Portopulmonary hypertension is a form of Group 1 Pulmonary Arterial Hypertension associated with liver disease and portal hypertension. The exact etiology of PPH is unknown, but the most widely accepted hypothesis is that vasoconstrictive mediators, which are typically metabolized by the liver, can reach the pulmonary circulation. This in combination with high shear stress from hyperdynamic circulatory state cause periarteriolar smooth muscle fibrosis and vasoconstriction [3, 6]. Diagnosis of PPH requires the presence of portal hypertension and excluding alternative causes of pulmonary arterial hypertension. Medical treatment of PPH includes pulmonary vasodilators including endothelin receptor antagonists, phosphodiesterase type-5 inhibitors, and prostanoids, although only one randomized clinical trial has specifically evaluated these therapies in patients with PPH [4, 7]. The only cure for PPH is liver transplant. Those with mPAP >35 mmHg must be treated with pulmonary vasodilators to decrease mPAP and pulmonary vascular resistance (PVR) before transplant, although some recent studies suggest that transplant should be considered when mPAP > 35 mmHg if there is a normal PVR [6]. Those patients diagnosed late in the disease course cannot achieve the hemodynamic requirements for liver transplant. POCUS was essential in this case to make a timely diagnosis. 

Several findings on POCUS expedited the diagnosis of PPH. Ultrasound assessment of the IVC can indirectly estimate the CVP which can further differentiate disease etiology [2, 8]. In the current case, ultrasound of the IVC identified a dilated IVC with limited respiratory variation, which is important for differentiating hepatic from cardiac ascites. In patients with cirrhosis, the IVC is typically small and collapsible because of reduced intravascular volume due to hypoalbuminemia. Additionally, tense ascites can compress the IVC. In contrast, cardiac ascites causes a distended IVC suggestive of increased right atrial pressure [2].

When IVC visualization suggested cardiac ascites, cardiac ultrasound of the right ventricular size, shape, and contractility then revealed several features suggestive of right ventricular dysfunction. In a structurally normal heart, the POCUS Rule-of-Thirds states at the right ventricle, the aortic outflow tract, and left atrium should all be the same size in the parasternal long axis view [2]. This case demonstrates that the right ventricle is volume overload because it is larger than the aortic outflow tract and left atrium (Video 3A). In the parasternal short axis view, the left ventricle is normally circular. When increased pressure in the right ventricle compresses the septum, the left ventricle takes on a D shape especially during diastole (Video 3B) [9]. Chronic pulmonary hypertension is often associated with mild to moderate pericardial effusions as was seen in this case (Video 3A,B) [10]. 

## Conclusions

Point-of-care ultrasound can be used in the workup of ascites to recognize cardiac etiologies. For many diseases, including PPH, timely diagnosis can maximize treatment options. Use of POCUS can narrow broad differentials and aid rapid diagnosis. Future studies should assess the impact of POCUS on subsequent diagnostic testing, accuracy, and cost.
